# Circulating Biomarkers of Accelerated Sarcopenia in Respiratory Diseases

**DOI:** 10.3390/biology9100322

**Published:** 2020-10-03

**Authors:** Rizwan Qaisar, Asima Karim, Tahir Muhammad, Islam Shah

**Affiliations:** 1 Department of Basic Medical Sciences, College of Medicine, University of Sharjah, Sharjah 27272, UAE; akarim@sharjah.ac.ae; 2 Department of Physiology and Cell Biology, University of Health Sciences, Lahore 54600, Pakistan; 3 Department of Biochemistry, Gomal Medical College, Dera Ismail Khan 29050, Pakistan; drtahir82@gmail.com; 4 Department of Cardiology, Al Qassimi Hospital, Sharjah 27272, UAE; islam.shah@moh.gov.ae

**Keywords:** sarcopenia, Dkk-3, CAF22, miRs, COPD, asthma, tuberculosis

## Abstract

**Simple Summary:**

As we grow older, our muscles become smaller and weaker, a condition called sarcopenia. Several lung diseases can further worsen sarcopenia. Among them, COPD, asthma and tuberculosis are well-recognized causes of muscle loss. However, it is difficult and time-consuming to assess muscle health in elderly people with lung diseases. Here, we aimed to overcome this problem by measuring the blood levels of specific molecules that are related to muscle size and strength. We first show that elderly people with lung diseases have a greater degree of sarcopenia than healthy people. We then show that the blood levels of certain molecules (Dkk-3, CAF22, microRNAs) have varying degrees of associations with muscle size and strength in these patients. Thus, we propose that these molecules can be useful in assessing muscle health and the physical capacity of the elderly with lung diseases. Our findings have clinical applications since the quality and/or quantity of muscle tissues decide everyday lifestyle in the elderly, such as walking, lifting from chair and going to the bathroom etc.

**Abstract:**

Skeletal muscle dysfunction is a critical finding in many respiratory diseases. However, a definitive biomarker to assess muscle decline in respiratory diseases is not known. We analyzed the association of plasma levels of glycoprotein Dickkopf-3 (Dkk-3), c-terminal agrin fragment-22 (CAF22) and microRNAs miR-21, miR-134a, miR-133 and miR-206 with hand-grip strength (HGS) and appendicular skeletal mass index (ASMI) in male, 54–73-year-old patients with chronic obstructive pulmonary diseases (COPD), asthma or pulmonary TB (*n* = 83–101/group). Patients with respiratory diseases showed a reduction in HGS and gait speed, while a reduction in ASMI was only found in patients with pulmonary TB. Among the sarcopenia indexes, HGS showed the strongest correlation with plasma CAF22, miR-21 and miR-206 levels while ASMI showed the strongest correlation with Dkk-3 and miR-133 in respiratory diseases. We found a modest-to-significant increase in the plasma markers of inflammation, oxidative stress and muscle damage, which had varying degrees of correlations with Dkk-3, CAF22 and selected micro RNAs (miRs) in respiratory diseases. Taken together, our data show that plasma levels of Dkk-3, CAF22 and selected miRs can be useful tools to assess accelerated sarcopenia phenotype in the elderly with respiratory diseases.

## 1. Introduction

Sarcopenia or age-related muscle loss is associated with poor health quality and functional dependence in the elderly. Additionally, there is a loss of muscle force-generating capacity independent of the muscle size, called dynapenia. The evaluation of sarcopenia included the assessments of muscle mass, dynapenia and physical performance [1]. Hand-grip strength (HGS) is often used to evaluate dynapenia as low HGS is a robust indicator of muscle wasting and physical dependence in aging. Reduction in HGS has been employed as a diagnostic marker of sarcopenia and disease severity in multiple age-related pathologies [2]. Muscle mass is evaluated with bioelectrical impedance analysis (BIA) in a user-friendly and cost-effective manner [3] while the reduced gait speed is a measure of compromised physical capacity in aging [4]. HGS, BIA and gait speed have been used as the predictors of pulmonary function in health and disease [5,6,7].

Chronic obstructive pulmonary diseases (COPD), asthma and pulmonary tuberculosis are among the lung diseases with systemic manifestations and can affect multiple systems including skeletal muscle. These diseases can result in systemic inflammation, poor oxygenation and oxidative stress [8,9], which can further exacerbate the age-related skeletal muscle detriment. Furthermore, the compromised functional capacity and exercise intolerance in patients with respiratory diseases also contribute to sarcopenia phenotype in the elderly. The accelerated sarcopenia in these diseases can further exacerbate muscle and respiratory decline and requires rigorous characterization for effective interventions. However, the techniques to evaluate muscle wasting are costly, time-consuming and may require radiation exposure, warranting the necessity of using plasma biomarkers as evaluation tools of sarcopenia. 

Several circulating molecules and parameters have been proposed as the potential biomarkers of sarcopenia. Among them, the c-reactive protein (CRP) and the erythrocyte sedimentation rate (ESR) are indicative of systemic inflammation, which is a frequent finding in aging [10]. However, due to their non-specificity to skeletal muscle, the circulating levels of these biomarkers may reflect the generalized health rather than the health of skeletal muscle. These findings necessitate the investigation of muscle-specific biomarkers such as myokines, as potential tools to assess sarcopenia. Among various myokines, irisin has emerged as a novel biomarker of muscle atrophy and weakness in debilitating conditions such as sarcopenia [11]. However, given the complex pathophysiology of sarcopenia, multiple biomarkers are required to accurately represent the muscle status in aging [12]. To date, no plasma biomarker is known to predict muscle quality and/or quantity in addition to well-recognized spirometry decline in respiratory diseases. Further, the ability of these biomarkers to independently predict functional capacity including the step count and gait speed is not known. 

Several molecular defects account for age-related muscle detriment, including deterioration of the neuromuscular junction (NMJ). In a healthy muscle, axon terminals maintain NMJ integrity by secreting agrin which helps in the aggregation of acetylcholine receptors on the synaptic site. Multiple catabolic conditions can result in proteolytic cleavage of agrin into its c-terminal agrin fragments (CAF), which cause destabilization of NMJ and lead to muscle wasting and weakness. C-terminal agrin fragment-22 (CAF22) is a smaller fragment of CAFs and its circulating levels are associated with the NMJ degeneration and muscle defect in sarcopenia [13] and other catabolic conditions due to heart failure and stroke [14,15]. However, the association of plasma CAF22 with muscle wasting in the respiratory diseases is not well recognized despite the evidence that smoking and other conditions of lung injury can result in the destabilization of NMJs [16].

Glycoprotein dickkopf-3 (Dkk-3) is a secreted glycoprotein belonging to the dickkopf family of proteins that are involved in the suppression of Wnt signaling. Recent results show that Dkk-3 expression in the skeletal muscle and plasma is increased in age-related muscle loss [17]. A direct causality between Dkk-3 expression and sarcopenia phenotype has been suggested as young mice with Dkk-3 overexpression mimic early sarcopenia phenotype while reducing Dkk-3 expression in aged mice partially offsets age-related muscle wasting [17]. These results suggest the diagnostic and prognostic significance of Dkk-3 in the characterization of sarcopenia. 

Micro RNAs (miRs) are small non-coding RNAs that control the post-transcriptional regulation of gene expression. Several studies appreciate the roles of miRs in regulating skeletal muscle health [18]. The expression of circulating miRs is altered in many muscle diseases including sarcopenia [19]. Several miRs have been proposed as potential biomarkers of sarcopenia. Among them, circulating miR-21, miR-134a, miR-133 and miR-206 have diagnostic and/or prognostic potential due to their correlations with physical capacity [20]. However, their associations with indexes of sarcopenia in respiratory diseases are not known.

We aimed to analyze the diagnostic properties of plasma Dkk-3, CAF22 and selected miRs to evaluate skeletal muscle detriment in COPD, asthma and pulmonary TB. We hypothesized that the altered levels of circulating biomarkers will be useful in predicting reduced HGS, muscle mass and the measures of exercise capacity in respiratory diseases. We tested this hypothesis by analyzing biological samples and collecting clinical and anthropometric data from a heterogeneous cohort of patients with COPD, asthma and pulmonary TB. 

## 2. Methods

### 2.1. Study Design and Participants 

Patients and the healthy participants were recruited after approvals by the regional ethical committee at the selected teaching hospitals of the University of Health Sciences, Lahore and Gomal Medical College, Dera Ismail Khan. Anthropometric data, plasma collection and measurements of body composition and handgrip strength were performed. Participants were divided into healthy controls (*n* = 101), COPD (*n* = 100), asthma (*n* = 87) and pulmonary TB (*n* = 83) groups. All participants were male, 54–73 years of age, with complete data from clinical examination, laboratory investigation, spirometry and HGS measurements. Written informed consent was obtained from all the study participants. The study population was selected from a large cohort of healthy controls and patients with respiratory diseases through simple random sampling to avoid selection bias. COPD was defined as forced expiratory volume in 1 s (FEV1)%/forced vital capacity (FVC) < 0.7 with persistent respiratory symptoms according to the GOLD guidelines [21]. Diagnosis of asthma and pulmonary TB was based on clinical features, laboratory investigations and/or spirometry as described elsewhere [22,23]. Subjects with the stable phenotype were included while those with the unstable phenotype (infection, exacerbation and/or hospitalization in the past month), arthritis, myopathies and neurological diseases were excluded. Subjects with higher plasma urea and/or creatinine were also excluded due to an independent association between plasma CAF22 levels and kidney function [15]. Body mass index (BMI) was calculated as kg/m^2^. Appendicular skeletal muscle mass (ASM) and fat mass were calculated with the bioelectrical impedance analysis scale (RENPHO, Dubai, UAE) as described previously [24]. ASM was divided by body area to get the appendicular skeletal muscle mass index (ASMI). This study was conducted in accordance with the declaration of Helsinki [25].

### 2.2. Hand-Grip Strength 

Hand-grip strength was measured using a digital handgrip dynamometer (CAMRY, South El Monte, CA, USA) as described before [26]. The participants were instructed to sit down with their elbows flexed at an angle of 90◦ with the dynamometer in hand in the supine position. The participants were then asked to squeeze the dynamometer with maximal strength in a smooth manner without rapid jerking or wrenching. No other body movement was allowed during the procedure. Three attempts were performed with each hand with a 60-s rest between each attempt and the highest value was recorded for analysis, as described by us previously [24]. 

### 2.3. Spirometry and Pulse Oximetry

The FEV1 and FVC were measured using a portable spirometer (Contec SP10, Qinhuangdao, Hebei, China), according to standards set by the American Thoracic Society [27]. A commercially available pulse oximeter was used to measure SpO_2_in healthy controls and patients with respiratory diseases (Nellcor N-200, Hayward, California, CA, USA).

### 2.4. Measurement of Plasma Biomarkers

For analysis of circulating biomarkers, plasma samples were collected in the morning in fasting state and assayed using ELISA kits for Dkk-3 (Cat # ab100502, Abcam) and CAF22 (NTCAF, ELISA, Neurotune, Schlieren-Zurich, Switzerland) according to the manufacturer’s instructions [24].

### 2.5. Measurements of Plasma 8-Isoprostanes, C-Reactive Proteins (CRP) and Creatine Kinase (CK)

We used ELISA to measure 8-isoprostanes (Cayman Chemical, Ann Arbor, MI, USA) and CRP (R&D Systems, Minneapolis, MN, USA) levels and biochemical assays to measure creatine kinase levels.

### 2.6. Quantification of Circulating miRs

Bulge-Loop TM miRNA qPCR Primer Sets (RiboBio) were used to detect selected miRs expressions by qRT-PCRs with iTaqTM Universal SYBR Green Supermix (BIO-RAD) as described elsewhere [28]. Reverse transcription of the miRs into cDNA was achieved with the TaqMan microRNA reverse transcription kit (Thermo Fisher, Dubai, UAE) [29] and TaqMan microRNA assays specific for selected miRs (Applied Biosystems, Thermo Fisher, Dubai, UAE) according to the manufacturer’s recommendations. Owing to several PCR sessions to analyze high number of samples, we created a reference sample by pooling a fraction of all control and CHF samples. This reference sample was run in each PCR session to minimize the technical variability in our samples. All qRT-PCR reactions were performed in triplicate, and the signal was collected at the end of every cycle. All miRs expressions were calibrated against spike-in cel-miR-39, which lacks sequence homology to human miRs.

### 2.7. Statistical Analysis

Anthropometric measurements of the participants were presented using mean and standard deviation as data met the assumption for normality using a Chi-square normality test. Analysis of variance with Tukey’s post-hoc test was used to compare continuous variables and the chi-square test was used to determine categorical variables between the groups. Pearson correlation was employed to determine the strength of the relationships of HGS and ASMI with the plasma biomarkers. A *p*-value < 0.05 was considered to be statistically significant. GraphPadPrism (GraphPad software, San Diego, California, CA, USA) version 6.01 was used for all statistical analysis.

## 3. Results

### 3.1. Characteristics of the Participants

Basic characteristics of the study population are summarized in Table 1.

**Figure 1:**
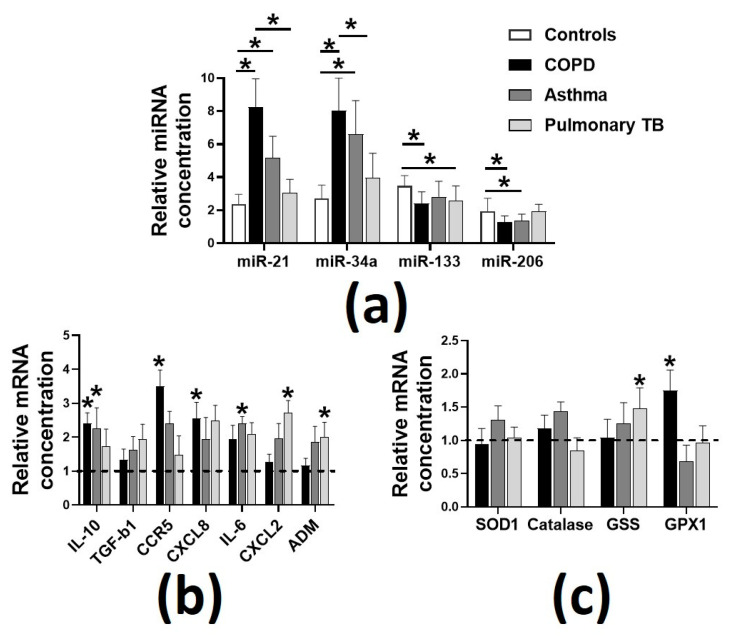
Plasma expressions of the selected micro RNAs (miRs) and the markers of inflammation and oxidative stress in the patients with respiratory diseases. (**a**), Sarcopenia in pulmonary diseases was associated with altered expressions of miR-21, miR-34a, miR-133 and miR-206; (**b**), elevated levels of the markers of inflammation; (**c**), relatively unperturbed levels of the markers of oxidative stress. Dotted line represents the normalized expressions in healthy controls. Values are expressed as mean ± SD, * *p* < 0.05 vs. healthy controls (*n* = 68–75 per group). Interleukin-10, IL-10; transforming growth factor-beta 1, TGF-b1; c-c motif chemokine receptor 5, CCR5; c-x-c motif chemokine ligand 8, CXCL8; interleukin-6, IL-6; c-x-c motif chemokine ligand 2, CXCL2; adrenomedullin, ADM; superoxide dismutase-1, SOD1; glutathione synthetase, GSS; glutathione peroxidase-1, GPX1.

Patients with pulmonary TB had significantly reduced BMI and percent fats than asthmatics and reduced ASMI compared to healthy controls. Respiratory diseases were associated with reduced HGS in COPD, asthma and pulmonary TB. After adjustment for ASMI, which can affect HGS [30], the normalized HGS was still significantly lower in participants with respiratory diseases (*p* < 0.05) than healthy controls. Gait speed and daily step count were used as the measures of functional capacity and were also significantly reduced (*p* < 0.05) in patients with respiratory diseases, when compared to healthy controls. Plasma Dkk-3 and CAF22 levels were higher in patients with COPD (≈24% and ≈93%, respectively, *p* < 0.05) and asthma (≈17% and ≈70%, respectively, *p* < 0.05), while the plasma CAF22 levels were elevated in patients with pulmonary TB (≈42%, *p* < 0.05) compared to healthy controls. We also report significantly higher levels of plasma miR-21 and miR-34a in the patients of respiratory diseases than healthy controls (Figure 1a). 

On the other hand, plasma miR-133 and miR-206 levels were downregulated in COPD, pulmonary TB (miR-133 only) and asthma (miR-206) (all *p* < 0.05). These changes were associated with perturbation in the circulating markers of inflammation (Figure 1b) and oxidative stress (Figure 1c). Specifically, COPD was associated with increased plasma expressions of interleukin-6 (IL-10), c-c motif chemokine receptor 5 (CCR5) and c-x-c motif chemokine ligand 8 (CXCL8). There was an increased plasma expression of IL-10 and IL-6 in asthma and of c-x-c motif chemokine ligand 2 (CXCL2) and adrenomedullin (ADM) in pulmonary TB. We also found increased plasma expression of glutathione synthetase (GSS) in pulmonary TB and glutathione Peroxidase-1 (GPX1) in patients with COPD.

### 3.2. Relationship of Plasma Biomarkers Levels with HGS and ASMI

**Figure 2:**
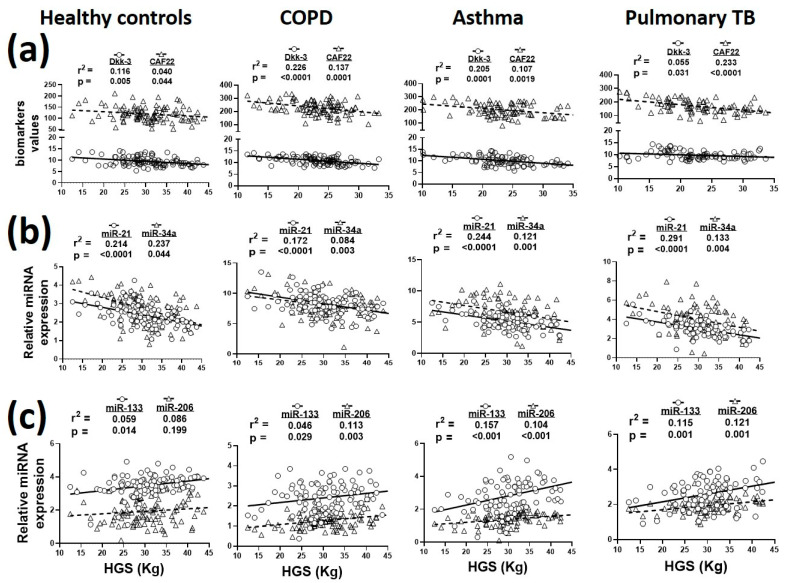
Relationship of plasma biomarkers with hand-grip strength (HGS) in healthy controls and patients with COPD, asthma and pulmonary TB. (**a**), Sarcopenia in respiratory diseases was associated with altered expressions of circulating Dkk-3, CAF22; (**b**), miR-21, miR-34a; (**c**), miR-133 and miR-206. *n* = 82–101 participants/group.

A total of 364 plasma samples from healthy controls and patients with respiratory diseases (*n* = 82–101/group) were analyzed for biomarker analysis. Seven samples were discarded because Dkk-3 and CAF22 were not detected in them. The associations of plasma biomarkers with HGS in respiratory diseases are summarized in Figure 2. Plasma Dkk-3 and CAF22 levels showed statistically significant associations with HGS in all study cohorts (Figure 2a). 

In general, plasma DKK-3 levels showed a stronger association with HGS in COPD and asthma, while CAF22 levels had the strongest association with HGS in pulmonary TB (Figure 2a). Among the selected miRs, miR-21 levels had the strongest association with HGS, followed by miR-34a and miR-206 (Figure 2b,c).

**Figure 3:**
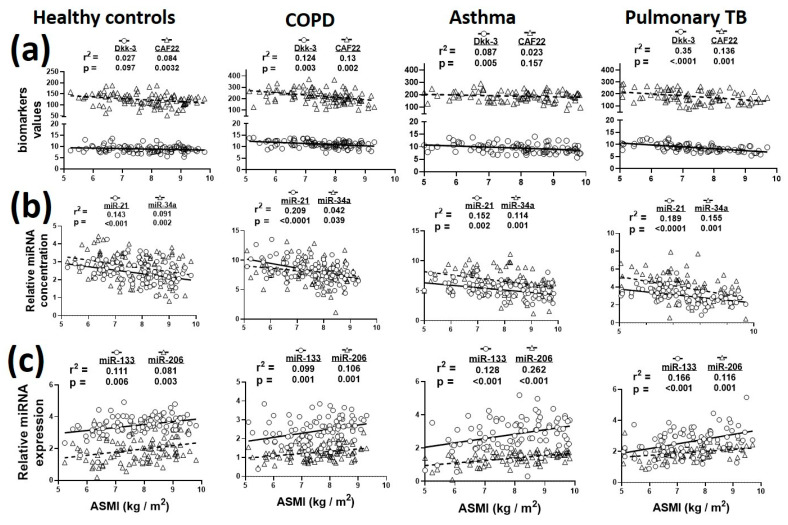
Relationship of plasma biomarkers with appendicular skeletal muscle index (ASMI) in healthy controls and patients with COPD, asthma and pulmonary TB. (**a**), Sarcopenia in respiratory diseases was associated with altered expressions of circulating Dkk-3, CAF22; (**b**), miR-21, miR-34a; (**c**), miR-133 and miR-206. *n* = 82–101 participants/group.

Since muscle wasting is a common consequence of several respiratory diseases, we next analyzed the correlations between plasma biomarkers and ASMI (Figure 3). In general, we did not find biologically significant associations of plasma DKK-3 and CAF22 with ASMI in respiratory diseases (Figure 3a). Among the miRs, miR-21 and miR-206 showed stronger associations with ASMI in various disease cohorts (Figure 3b,c). 

### 3.3. Correlation of Plasma Biomarkers with Gait Speed and Daily Steps Count

Since reduced gait speed and steps count indicate functional status in the elderly [31,32], we next evaluated the association of plasma biomarkers with gait speed and daily step count in the respiratory diseases (Table 2). Overall, we observed that plasma Dkk-3 and CAF22 levels are better predictors of gait speed and steps count than selected miRs. Among the miRs, miR-21 and miR-206 showed significant relations with steps count in some study cohorts while the association of miR-34a and miR-133 with gait speed and step count was modest (Table 2).

### 3.4. Relationships of Respiratory Diseases with Plasma Markers of Inflammation, Oxidative Stress and Muscle Damage

Due to the close association of respiratory diseases with muscle detriment [33], oxidative stress and inflammation [34], we next analyzed the associations of plasma biomarkers with 8-isoprostane (marker of oxidative stress), CRP (marker of inflammation) and creatine kinase (marker of muscle damage) in respiratory diseases (Table 3). We found a significant association of plasma 8-isoprostane levels with miR-133 and miR-206, and plasma CRP levels with miR-21, miR-34a, miR-133 and miR-206 in all study groups. On the other hand, plasma creatine kinase showed significant correlation with plasma CAF22, miR-133 and miR-206 in all study groups (Table 3). 

### 3.5. Correlation of Plasma Dkk-3, CAF22 and miRs with Each Other in Respiratory Diseases

Since selected biomarkers share multiple signaling pathways and target organs, we next evaluated the associations among the circulating Dkk-3, CAF22 and selected miRs in our study cohorts. We found significant associations among multiple biomarkers in various groups (Table 4). Of interest, plasma CAF22 levels showed significant associations with miR-21, miR-34a, miR-133 and miR-206 in most of the diseases. Among other biomarkers, a significant association between miR-21 and miR-34a was found in all diseases and healthy controls, while other biomarkers showed varying degrees of associations in respiratory diseases. 

### 3.6. Relationship of Hand-grip Strength with ASMI in Respiratory Diseases

**Figure 4:**
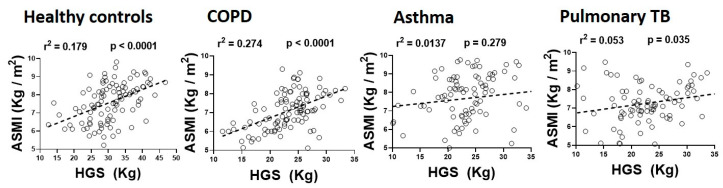
Relationship of appendicular skeletal muscle index (ASMI) with hand-grip strength (HGS) in healthy controls and patients with COPD, asthma and pulmonary TB (*n* = 82–101 participants/group).

We next investigated the association between ASMI and HGS in respiratory diseases. A robust positive association between ASMI and HGS was found in patients with COPD (r^2^ = 0.274, *p* < 0.001). We also found a modest but statistically significant association between ASMI and HGS in patients with pulmonary TB (r^2^ = 0.053, *p* < 0.05) but not in the asthmatic patients (r^2^ = 0.0137, *p* = 0.279), as depicted in Figure 4. 

## 4. Discussion

In this study, we investigated the direct correlation of circulating levels of Dkk-3, CAF22 and selected miRs with sarcopenia in respiratory diseases. In summary, we show that these circulating biomarkers can predict indexes of accelerated sarcopenia in respiratory diseases to varying degrees. We found a significant association of these biomarkers with HGS, ASMI and gait speed in COPD, asthma and pulmonary TB, which suggests that Dkk-3, CAF22 and selected miRs can be useful biomarkers of sarcopenia in respiratory diseases. 

The complexity of muscle aging is a major challenge to investigating clinically relevant biomarkers of sarcopenia. Respiratory diseases further exacerbate age-related muscle decline and the search for circulating biomarkers to accurately predict muscle mass and strength in such conditions remain elusive. These diseases also result in increased systemic inflammation and oxidative stress, which can independently contribute to the loss of skeletal muscle mass and strength irrespective of the spirometry decline. This is evident by a significant association of the muscle atrophy and weakness with the markers of inflammation and oxidative stress in aging [10]. In support of this, we found higher expression of the inflammation and oxidative stress markers in all three pulmonary diseases. However, these markers are non-specific and represent generalized health rather than the health status of skeletal muscle and/or lungs. Dkk-3 offers an advantage over these biomarkers because of its expression in skeletal muscle and association with systemic inflammation [17]. The circulating Dkk-3 levels are increased in aging and in conditions involving the cellular senescence [35] which implies that it has a role in the age-associated disorders. However, a direct correlation of plasma Dkk-3 with muscle quality and/or quantity in sarcopenia has not been recognized before. We found a significant association of circulating Dkk-3 with HGS, which shows that Dkk-3 can be a useful tool to assess dynapenia in the elderly population with respiratory diseases. We did not investigate the potential mechanism(s) by which Dkk-3 can contribute to sarcopenia in the elderly with respiratory diseases. At the molecular level, Dkk-3 inhibits Wnt signaling and drives muscle loss by activating Fbxo32 and Trim63. Interestingly, Wnt signaling is suppressed in the airway epithelia of smokers and patients with COPD [36] and can potentially contribute to an increase in plasma Dkk-3 levels and muscle detriment in these patients. Among the three respiratory diseases, the strongest correlation between plasma Dkk-3 and ASMI was found in patients with pulmonary TB. It must be noted that compared to COPD and asthma, pulmonary TB leads to a relatively accelerated muscle loss in a short span of time [37]. Thus, it is possible that Dkk-3 activation has a greater contribution to relatively accelerated sarcopenia in the pulmonary TB compared to atrophy in more chronic diseases such as COPD and asthma. In support of this, Dkk-3 overexpression in the cultured myotubes leads to accelerated muscle atrophy [17]. However, not all types of muscle atrophies are Dkk-3 dependent. For example, atrophies due to starvation and cancer cachexia can occur independently of Dkk-3 activation [17]. Thus, while we confirm and extend the usefulness of Dkk-3 as a biomarker of sarcopenia, it is possible that it might not accurately predict the accelerated sarcopenia phenotype in all age-related systemic diseases. For example, Dkk-3 expression is reduced in several cancers and shows no correlation with the muscle atrophy in cancer cachexia, which occurs independently of changes in Wnt signaling [17].

NMJ degeneration is a characteristic feature of aging muscle. This is partly due to loss of protective myokines such as irisin, which protect skeletal muscle against denervation-induced atrophy [38]. Indeed, aging is associated with reduced circulating levels of irisin [11], which can potentially contribute to NMJ fragmentation and muscle denervation. In support of this, we found higher levels of plasma CAF22 in all three respiratory diseases, which indicate degeneration of NMJs in the skeletal muscles from these patients. Our previous work shows that the NMJ disruption can induce muscle atrophy and weakness [39], which can potentially indicate a negative association between the plasma CAF22 levels, the byproduct of NMJ disruption, and muscle impediment. Higher circulating CAF22 levels have been reported in multiple muscle-wasting conditions in sarcopenia [13], stroke [15] and heart failure [14]. Additionally, smoking, which is a common cause of several respiratory diseases, is recently shown to induce the degeneration of NMJs [16]. A large proportion of our patients with COPD and asthma had smoking history and it is possible that elevated plasma CAF22 levels in these patients are at least partly due to the smoking-induced NMJ disruption. Additionally, COPD can also induce structural and functional defects in NMJs independent of smoking [16,40,41], which can also contribute to the increased circulating CAF22 levels and the muscle loss in COPD. 

We found varying degrees of associations among circulating biomarkers. Of note is the robust association of CAF22 with plasma miRs levels. All the miRs investigated by us have some degree of contribution in NMJ regulation and maintenance [42,43,44]. For example, miR-206 is involved in NMJ repair following nerve injury, and this action is either mediated alone or in combination with miR-133 [42]. Similarly, miR-34a regulated synaptogenesis by mediating the interaction between presynaptic and postsynaptic terminals [44]. The association of these miRs with CAF22 shows that they likely share signaling pathways involved in NMJ disruption in aging and respiratory diseases. 

Among the miRs, miR-21, miR-206 and miR-133 emerge as potential candidate biomarkers of sarcopenia indexes. The diagnostic potential of these miRs in skeletal muscle diseases has been described previously [19,45]. Among the selected miRs, miR-206 and miR-133 are primarily expressed in skeletal muscle, while miR-34a and miR-21 are expressed by multiple tissues [46]. Given the multi-system effects of COPD, asthma and pulmonary TB, it is possible that the altered plasma levels of these miRs represent a generalized clinical state of the patient in addition to skeletal muscle detriment. Further, it is not known whether the contribution of skeletal muscle to plasma miRs is due to passive release from damaged muscle fibers or involve active mechanisms. However, a significant correlation of miR-21, miR-133 and miR-206 miRs with plasma creatine kinase levels show that the myofibers damage at least partly contributes to circulating miRs levels. 

A correlation between ASMI and HGS has recently been described in sarcopenia due to cancer-cachexia [47]. In agreement with those results, we found a significant association between muscle atrophy and dynapenia in COPD participants. However, the coupling between muscle quantity and quality was lost in patients with asthma and pulmonary TB. We do not know the exact cause of this discrepancy. However, ASMI is a quantitative measure and does not consider the quality of the skeletal muscle. Multiple factors including edema, fat infiltration and disruption of the intracellular contractile machinery can reduce muscle quality without significantly affecting the muscle quantity [48]. Further studies are required to analyze muscle biopsies for comparing muscle quality to quantity. 

Limitations of this study include the heterogeneity of individual cohorts. We tried to minimize this by recruiting participants from a relatively homogenous population from a well-defined geographical region. However, like any cohort study, the selective survival of the participants before their recruitments into this study should be considered. The sample size for each group is modest for drawing stronger conclusions. The age groups of the participants may not represent the advanced sarcopenia in the relatively elderly Western population. The quadriceps muscles strength was not measured, which has a greater influence on the quality of life in sarcopenia than the HGS [49]. 

## 5. Conclusions

Taken together, we have shown that the expressions of circulating Dkk-3, CAF22 and selected miRs have varying degrees of statistical relations with the indexes of sarcopenia and physical capacity in the elderly with respiratory diseases. We also report a coupling between the muscle mass and strength in COPD but not in asthma and pulmonary TB. These findings elucidate the biomarker potential of the circulating Dkk-3, CAF22 and selected miRs in assessing muscle health and functional capacity in the elderly with respiratory diseases. However, further studies with the mechanistic and temporal approaches are required before establishing the biological significance of these findings.

## Figures and Tables

**Table 1:** Body composition, physical parameters and plasma biomarkers in healthy controls patients with chronic obstructive pulmonary diseases (COPD), asthma and pulmonary TB. Values are expressed as mean ± SD; one-way analysis of variance. * *p* < 0.05 vs. healthy controls; # *p* < 0.05 vs. COPD patients; ƙ *p* < 0.05 vs. asthma patients (*n* = 83–101 per group).

	Healthy Controls	COPD	Asthma	Pulmonary TB
Age at baseline (years)	65.1 ± 5.5	68.1 ± 6.5	58.7 ± 4.2	62.8 ± 5.1
Number of Participants	101	100	87	83
Body composition				
BMI (Kg/m^2^)	25.1 ± 4.4	25.9 ± 4.1	27.2 ± 3.5	23.1 ± 4.2 ƙ
ASM (Kg)	23.8 ± 4.7	22.1 ± 4.1	23.2 ± 3.8	22 ± 3.4
ASMI (Kg/m^2^)	8.3 ± 1.5	7.8 ± 1.35	7.6 ± 1.3	7.1 ± 1.4 *
Percent fat	37 ± 4.1	36.4 ± 4.6	42.1 ± 5.1	34.2 ± 4.6 ƙ
Physical Parameters				
HGS (kg)	29.4 ± 6.4	23.1 ± 4.1 *	24.1 ± 4.3 *	22.4 ± 5.3 *
HGS/ASM	1.23 ± 0.31	1.06 ± 0.23 *	1.03 ± 0.21 *	0.99 ± 0.26 *#
4-Meter Gait Speed (m/s)	1.13 ± 0.27	0.94 ± 0.29 *	0.98 ± 0.33 *	1.01 ± 0.22 *
Daily steps count	7171 ± 1,609	4103 ± 1,276 *	3101 ± 1,133 *#	3257 ± 855 *#
Spirometry and oxygen saturation				
FEV1% predicted	95.55 ± 5.7	55.68 ± 5.5 *	49.7 ± 4.6 *	59.9 ± 5.4 *
PEFR% predicted	90.91 ± 5.5	69.48 ± 5.5 *	61.51 ± 5.7 *	63.33 ± 5.9 *
SpO_2_	98.1 ± 2.3	93.2 ± 2.5 *	92.1 ± 2.2 *	94.4 ± 1.8 *
Proportion of smokers, *n* (%)	12 (12.1%)	39 (39%)	33 (37%)	8 (9.6%)
Plasma biomarkers				
Dkk-3 (ng/ul)	8.72 ± 1.3	10.87 ± 1.4 *	10.22 ± 1.5 *	9.41 ± 1.3
CAF22 (pM)	118.7 ± 24.7	228.6 ± 34.7 *	201.6 ± 39.2 *	168.7 ± 41.4 *#
8-isoprostanes (pg/mL)	53.9 ± 11.7	81.3 ± 12.4 *	93.2 ± 16.6 *	69.3 ± 10.4 *
CRP(mg/dl)	0.170 ± 0.04	0.292 ± 0.07 *	0.268 ± 0.05 *	0.263 ± 0.04 *
Creatine kinase (IU/L)	183.5 ± 27.3	293.3 ± 39.7 *	244 ± 35.4 *	318.4 ± 58.3 *

Note: BMI, body mass index; ASM, appendicular skeletal mass; ASMI, appendicular skeletal mass index; HGS, hand-grip strength; FEV1, forced expiratory volume in 1 s; PEFR, peak expiratory flow rate; Dkk-3, Dickkopf-3; CAF22, c-terminal agrin fragment-22; CRP, c-reactive protein.

**Table 2:** Correlations coefficients of plasma biomarkers with gait speed and daily steps count in healthy controls and patients with COPD, asthma and pulmonary TB.

		Healthy Controls	COPD	Asthma	Pulmonary TB
Dkk-3	Gait speed Step count	0.123 * 0.166 *	0.274 ** 0.314 **	0.199 * 0.093	0.099 0.081
CAF22	Gait speed Step count	0.111 * 0.093	0.198 * 0.023 *	0.118 * 0.323 **	0.051 0.128 *
miR-21	Gait speed Step count	0.071 0.068	0.095 * 0.118 *	0.029 0.217 **	0.058 0.079
miR-34a	Gait speed Step count	0.083 * 0.028	0.048 0.073 *	0.051 0.063	0.069 0.093 *
miR-133	Gait speed Step count	0.063 0.066	0.073 0.051	0.043 0.89 *	0.066 0.052
miR-206	Gait speed Step count	0.046 0.071 *	0.061 0.058	0.083 * 0.128 *	0.066 0.109 *

Dkk-3, Dickkopf-3; CAF22, c-terminal agrin fragment-22; *n* = 82–101 per group; * *p* < 0.05, ** *p* < 0.001.

**Table 3:** Correlations coefficients of circulating biomarkers with plasma 8-isoprostanes, CRP and creatine kinase levels in healthy controls and patients with COPD, asthma and pulmonary TB.

		Healthy Controls	COPD	Asthma	Pulmonary TB
Dkk-3	8-isoprostanes Plasma CRP Creatine kinase	0.073 0.059 0.031	0.103 * 0.086 0.061	0.073 0.063 0.083	0.096 * 0.105 * 0.141 *
CAF22	8-isoprostanes Plasma CRP Creatine kinase	0.064 0.083 0.129 *	0.055 0.122 * 0.184 *	0.074 0.116 * 0.163 *	0.092 * 0.051 0.138 *
miR-21	8-isoprostanes Plasma CRP Creatine kinase	0.114 * 0.094 * 0.239 **	0.071 0.169 * 0.107 *	0.131 * 0.183 * 0.162 *	0.057 0.138 * 0.174 *
miR-34a	8-isoprostanes Plasma CRP Creatine kinase	0.133 * 0.196 * 0.021	0.147 * 0.203 ** 0.069	0.112 * 0.158 * 0.081 *	0.052 0.103 * 0.088 *
miR-133	8-isoprostanes Plasma CRP Creatine kinase	0.129 * 0.116 * 0.107 *	0.181 ** 0.085 * 0.129 *	0.108 * 0.104 * 0.156 **	0.144 * 0.096 * 0.084 *
miR-206	8-isoprostanes Plasma CRP Creatine kinase	0.097 * 0.133 * 0.169 *	0.134 * 0.173 ** 0.158 *	0.085 * 0.078 * 0.147 *	0.139 * 0.088 * 0.121 *

*n* = 82–101 per group; * *p* < 0.05, ** *p* < 0.001.

**Table 4:** Correlations coefficients of the circulating biomarkers in healthy controls and patients with COPD, asthma and pulmonary TB.

	CAF22	miR-21	miR-34a	miR-133	miR-206
Dkk-3	0.086 0.251 * 0.194 * 0.109	0.106 0.185 * 0.127 0.195 **	0.117 * 0.229 ** 0.204 * 0.185 *	0.048 0.128 0.108 0.156 *	0.093 0.105 0.134 * 0.126
CAF22		0.122 0.361 ** 0.285 ** 0.176 *	0.118 * 0.172 * 0.189 ** 0.164 *	0.096 0.145 * 0.159 * 0.183 *	0.071 0.243 ** 0.286 ** 0.353 **
miR-21			0.194 * 0.265 ** 0.384 ** 0.319 **	0.077 0.139 * 0.128 0.121	0.104 0.055 0.188 * 0.201 **
miR-34a				0.067 0.122 0.106 0.186 **	0.185 * 0.205 * 0.238 ** 0.108
miR-133					0.111 * 0.104 0.057 0.069

Dkk-3, Dickkopf-3; CAF22, c-terminal agrin fragment-22; A. healthy controls; B. COPD; C. asthma; D. pulmonary TB; *n* = 82–101 per group; * *p* < 0.05, ** *p* < 0.001.

## References

[1] Cruz-Jentoft A.J., Baeyens J.P., Bauer J.M., Boirie Y., Cederholm T., Landi F., Martin F.C., Michel J.P., Rolland Y., Schneider S.M. (2010). Sarcopenia: European consensus on definition and diagnosis: Report of the European Working Group on Sarcopenia in Older People. Age Ageing.

[2] Bae E.J., Park N.J., Sohn H.S., Kim Y.H. (2019). Handgrip Strength and All-Cause Mortality in Middle-Aged and Older Koreans. Int. J. Environ. Res. Public Health.

[3] Fujimoto K., Inage K., Eguchi Y., Orita S., Suzuki M., Kubota G., Sainoh T., Sato J., Shiga Y., Abe K. (2018). Use of Bioelectrical Impedance Analysis for the Measurement of Appendicular Skeletal Muscle Mass/Whole Fat Mass and Its Relevance in Assessing Osteoporosis among Patients with Low Back Pain: A Comparative Analysis Using Dual X-ray Absorptiometry. Asian Spine J..

[4] Harada H., Kai H., Shibata R., Niiyama H., Nishiyama Y., Murohara T., Yoshida N., Katoh A., Ikeda H. (2017). New diagnostic index for sarcopenia in patients with cardiovascular diseases. PLoS ONE.

[5] Han C.H., Chung J.H. (2018). Association between hand grip strength and spirometric parameters: Korean National health and Nutrition Examination Survey (KNHANES). J. Thorac. Dis..

[6] Hollander-Kraaijeveld F.M., Lindeman Y., de Roos N.M., Burghard M., van de Graaf E.A., Heijerman H.G.M. (2020). Non-fasting bioelectrical impedance analysis in cystic fibrosis: Implications for clinical practice and research. J. Cyst. Fibros..

[7] Kon S.S., Patel M.S., Canavan J.L., Clark A.L., Jones S.E., Nolan C.M., Cullinan P., Polkey M.I., Man W.D. (2013). Reliability and validity of 4-metre gait speed in COPD. Eur. Respir. J..

[8] Lee K.Y., Ito K., Maneechotesuwan K. (2016). Inflammation to Pulmonary Diseases. Mediat. Inflamm..

[9] Park H.S., Kim S.R., Lee Y.C. (2009). Impact of oxidative stress on lung diseases. Respirology.

[10] Can B., Kara O., Kizilarslanoglu M.C., Arik G., Aycicek G.S., Sumer F., Civelek R., Demirtas C., Ulger Z. (2017). Serum markers of inflammation and oxidative stress in sarcopenia. Aging Clin. Exp. Res..

[11] Chang J.S., Kim T.H., Nguyen T.T., Park K.S., Kim N., Kong I.D. (2017). Circulating irisin levels as a predictive biomarker for sarcopenia: A cross-sectional community-based study. Geriatr. Gerontol. Int..

[12] Kwak J.Y., Hwang H., Kim S.K., Choi J.Y., Lee S.M., Bang H., Kwon E.S., Lee K.P., Chung S.G., Kwon K.S. (2018). Prediction of sarcopenia using a combination of multiple serum biomarkers. Sci. Rep..

[13] Drey M., Sieber C.C., Bauer J.M., Uter W., Dahinden P., Fariello R.G., Vrijbloed J.W. (2013). C-terminal Agrin Fragment as a potential marker for sarcopenia caused by degeneration of the neuromuscular junction. Exp. Gerontol..

[14] Steinbeck L., Ebner N., Valentova M., Bekfani T., Elsner S., Dahinden P., Hettwer S., Scherbakov N., Schefold J.C., Sandek A. (2015). Detection of muscle wasting in patients with chronic heart failure using C-terminal agrin fragment: Results from the Studies Investigating Co-morbidities Aggravating Heart. Eur. J. Heart Fail..

[15] Scherbakov N., Knops M., Ebner N., Valentova M., Sandek A., Grittner U., Dahinden P., Hettwer S., Schefold J.C., von Haehling S. (2016). Evaluation of C-terminal Agrin Fragment as a marker of muscle wasting in patients after acute stroke during early rehabilitation. J. Cachexia Sarcopenia Muscle.

[16] Kapchinsky S., Vuda M., Miguez K., Elkrief D., de Souza A.R., Baglole C.J., Aare S., MacMillan N.J., Baril J., Rozakis P. (2018). Smoke-induced neuromuscular junction degeneration precedes the fibre type shift and atrophy in chronic obstructive pulmonary disease. J. Physiol..

[17] Yin J., Yang L., Xie Y., Liu Y., Li S., Yang W., Xu B., Ji H., Ding L., Wang K. (2018). Dkk3 dependent transcriptional regulation controls age related skeletal muscle atrophy. Nat. Commun..

[18] Diniz G.P., Wang D.Z. (2016). Regulation of Skeletal Muscle by microRNAs. Compr. Physiol..

[19] Yanai K., Kaneko S., Ishii H., Aomatsu A., Ito K., Hirai K., Ookawara S., Ishibashi K., Morishita Y. (2020). MicroRNAs in Sarcopenia: A Systematic Review. Front. Med. (Lausanne).

[20] Zhang T., Brinkley T.E., Liu K., Feng X., Marsh A.P., Kritchevsky S., Zhou X., Nicklas B.J. (2017). Circulating MiRNAs as biomarkers of gait speed responses to aerobic exercise training in obese older adults. Aging.

[21] Mirza S., Clay R.D., Koslow M.A., Scanlon P.D. (2018). COPD Guidelines: A Review of the 2018 GOLD Report. Mayo Clin. Proc..

[22] Bateman E.D., Hurd S.S., Barnes P.J., Bousquet J., Drazen J.M., FitzGerald J.M., Gibson P., Ohta K., O’Byrne P., Pedersen S.E. (2008). Global strategy for asthma management and prevention: GINA executive summary. Eur. Respir. J..

[23] Ryu Y.J. (2015). Diagnosis of pulmonary tuberculosis: Recent advances and diagnostic algorithms. Tuberc. Respir. Dis..

[24] Qaisar R., Karim A., Muhammad T. (2020). Plasma CAF22 Levels as a Useful Predictor of Muscle Health in Patients with Chronic Obstructive Pulmonary Disease. Biology.

[25] World Medical A. (2013). World Medical Association Declaration of Helsinki: Ethical principles for medical research involving human subjects. JAMA.

[26] Qaisar R., Karim A., Muhammad T. (2020). Circulating Biomarkers of Handgrip Strength and Lung Function in Chronic Obstructive Pulmonary Disease. Int. J. Chron. Obstruct. Pulmon. Dis..

[27] Culver B.H., Graham B.L., Coates A.L., Wanger J., Berry C.E., Clarke P.K., Hallstrand T.S., Hankinson J.L., Kaminsky D.A., MacIntyre N.R. (2017). Recommendations for a Standardized Pulmonary Function Report. An Official American Thoracic Society Technical Statement. Am. J. Respir. Crit. Care Med..

[28] He N., Zhang Y.L., Zhang Y., Feng B., Zheng Z., Wang D., Zhang S., Guo Q., Ye H. (2020). Circulating MicroRNAs in Plasma Decrease in Response to Sarcopenia in the Elderly. Front. Genet..

[29] Qaisar R., Bhaskaran S., Ranjit R., Sataranatarajan K., Premkumar P., Huseman K., Van Remmen H. (2019). Restoration of SERCA ATPase prevents oxidative stress-related muscle atrophy and weakness. Redox Biol..

[30] Bandyopadhyay A. (2008). Body composition and hand grip strength in male brick-field workers. Malays. J. Med. Sci..

[31] Karpman C., Benzo R. (2014). Gait speed as a measure of functional status in COPD patients. Int. J. Chron. Obstruct. Pulmon. Dis..

[32] Park H., Park S., Shephard R.J., Aoyagi Y. (2010). Yearlong physical activity and sarcopenia in older adults: The Nakanojo Study. Eur. J. Appl. Physiol..

[33] Jaitovich A., Barreiro E. (2018). Skeletal Muscle Dysfunction in Chronic Obstructive Pulmonary Disease. What We Know and Can Do for Our Patients. Am. J. Respir. Crit. Care Med..

[34] Rahman I., Adcock I.M. (2006). Oxidative stress and redox regulation of lung inflammation in COPD. Eur. Respir. J..

[35] Zenzmaier C., Sklepos L., Berger P. (2008). Increase of Dkk-3 blood plasma levels in the elderly. Exp. Gerontol..

[36] Wang R., Ahmed J., Wang G., Hassan I., Strulovici-Barel Y., Hackett N.R., Crystal R.G. (2011). Down-regulation of the canonical Wnt beta-catenin pathway in the airway epithelium of healthy smokers and smokers with COPD. PLoS ONE.

[37] Paton N.I., Ng Y.M. (2006). Body composition studies in patients with wasting associated with tuberculosis. Nutrition.

[38] Reza M.M., Subramaniyam N., Sim C.M., Ge X., Sathiakumar D., McFarlane C., Sharma M., Kambadur R. (2017). Irisin is a pro-myogenic factor that induces skeletal muscle hypertrophy and rescues denervation-induced atrophy. Nat. Commun..

[39] Sataranatarajan K., Qaisar R., Davis C., Sakellariou G.K., Vasilaki A., Zhang Y., Liu Y., Bhaskaran S., McArdle A., Jackson M. (2015). Neuron specific reduction in CuZnSOD is not sufficient to initiate a full sarcopenia phenotype. Redox. Biol..

[40] Kaplan Y., Gulbas G., Ermis H., Kamisli O., Kamisli S., Ozcan C. (2012). Investigation of neuromuscular transmission in patients with chronic obstructive pulmonary disease: A preliminary report. Eur. Respir. J..

[41] Gulbas G., Kaplan Y., Kamisli O., Ermis H., Kamisli S., Ozcan C. (2013). Neuromuscular transmission in hypoxemic patients with chronic obstructive pulmonary disease. Respir. Physiol. Neurobiol..

[42] Valdez G., Heyer M.P., Feng G., Sanes J.R. (2014). The role of muscle microRNAs in repairing the neuromuscular junction. PLoS ONE.

[43] Punga A.R., Andersson M., Alimohammadi M., Punga T. (2015). Disease specific signature of circulating miR-150-5p and miR-21-5p in myasthenia gravis patients. J. Neurol Sci.

[44] McNeill E.M., Warinner C., Alkins S., Taylor A., Heggeness H., DeLuca T.F., Fulga T.A., Wall D.P., Griffith L.C., Van Vactor D. (2020). The conserved microRNA miR-34 regulates synaptogenesis via coordination of distinct mechanisms in presynaptic and postsynaptic cells. Nat. Commun..

[45] Alexander M.S., Kunkel L.M. (2015). Skeletal Muscle MicroRNAs: Their Diagnostic and Therapeutic Potential in Human Muscle Diseases. J. Neuromuscul. Dis..

[46] Ludwig N., Leidinger P., Becker K., Backes C., Fehlmann T., Pallasch C., Rheinheimer S., Meder B., Stahler C., Meese E. (2016). Distribution of miRNA expression across human tissues. Nucleic Acids Res..

[47] Moreau J., Ordan M.A., Barbe C., Mazza C., Perrier M., Botsen D., Brasseur M., Portefaix C., Renard Y., Talliere B. (2019). Correlation between muscle mass and handgrip strength in digestive cancer patients undergoing chemotherapy. Cancer Med..

[48] Lee K., Shin Y., Huh J., Sung Y.S., Lee I.S., Yoon K.H., Kim K.W. (2019). Recent Issues on Body Composition Imaging for Sarcopenia Evaluation. Korean J. Radiol..

[49] Harris-Love M.O., Benson K., Leasure E., Adams B., McIntosh V. (2018). The Influence of Upper and Lower Extremity Strength on Performance-Based Sarcopenia Assessment Tests. J. Funct. Morphol.Kinesiol..

